# Maternal DHA supplementation influences sex-specific disruption of placental gene expression following early prenatal stress

**DOI:** 10.1186/s13293-020-00356-x

**Published:** 2021-01-09

**Authors:** Eldin Jašarević, Patrick M. Hecht, Kevin L. Fritsche, David C. Geary, Rocío M. Rivera, David Q. Beversdorf

**Affiliations:** 1 Interdisciplinary Neuroscience Program, University of Missouri, One Hospital Drive, DC069.10, Columbia, MO 65211 USA; 2 Thompson Center for Autism and Neurodevelopmental Disorders, University of Missouri, One Hospital Drive, DC069.10, Columbia, MO 65211 USA; 3 Department of Nutrition and Exercise Physiology, University of Missouri, One Hospital Drive, DC069.10, Columbia, MO 65211 USA; 4 Department of Psychological Sciences, University of Missouri, One Hospital Drive, DC069.10, Columbia, MO 65211 USA; 5 Division of Animal Sciences, University of Missouri, One Hospital Drive, DC069.10, Columbia, MO 65211 USA; 6 Department of Radiology, University of Missouri, One Hospital Drive, DC069.10, Columbia, MO 65211 USA; 7 Department of Neurology, University of Missouri, One Hospital Drive, DC069.10, Columbia, MO 65211 USA

**Keywords:** Docosahexaenoic acid, Placenta, Maternal diet, Sex differences, Prenatal stress

## Abstract

Early life adversity is widely recognized as a key risk factor for early developmental perturbations and contributes to the presentation of neuropsychiatric disorders in adulthood. Neurodevelopmental disorders exhibit a strong sex bias in susceptibility, presentation, onset, and severity, although the underlying mechanisms conferring vulnerability are not well understood. Environmental perturbations during pregnancy, such as malnutrition or stress, have been associated with sex-specific reprogramming that contribute to increased disease risk in adulthood, whereby stress and nutritional insufficiency may be additive and further exacerbate poor offspring outcomes. To determine whether maternal supplementation of docosahexanoic acid (DHA) exerts an effect on offspring outcome following exposure to early prenatal stress (EPS), dams were fed a purified 10:1 omega-6/omega-3 diet supplemented with either 1.0% preformed DHA/kg feed weight (DHA-enriched) or no additional DHA (denoted as the control diet, CTL). Dams were administered chronic variable stress during the first week of pregnancy (embryonic day, E0.5–7.5), and developmental milestones were assessed at E 12.5. Exposure to early prenatal stress (EPS) decreased placenta and embryo weight in males, but not females, exposed to the CTL diet. DHA enrichment reversed the sex-specific decrease in placenta and embryo weight following EPS. Early prenatal exposure upregulated expression of genes associated with oxygen and nutrient transport, including hypoxia inducible factor 3α (HIF3α), peroxisome proliferator-activated receptor alpha (PPARα), and insulin-like growth binding factor 1 (IGFBP1), in the placenta of CTL diet males exposed to EPS. DHA enrichment in EPS-exposed animals abrogated the male-specific upregulation of PPARα, HIF3α, and IGFBP1. Taken together, these studies suggest that maternal dietary DHA enrichment may buffer against maternal stress programming of sex-specific outcomes during early development.

## Introduction

Environmental perturbations during pregnancy, such as stress and malnutrition, are key risk factors for neurodevelopmental and neuropsychiatric disorders [1, 2]. Epidemiological studies have shown that in utero exposure to infections, hypoxia, stress, and malnutrition during the first trimester predicted increased risk for schizophrenia in males, whereas chronic stress during the second and third trimesters predicted a higher incidence of autism spectrum disorders in boys [3–6]. Indeed, nearly all neurodevelopmental disorders exhibit sex differences in the presentation, age of onset, and treatment outcome [7, 8]. Parallel to epidemiological findings, exposure to chronic stress during the first week of pregnancy produced lasting effects in male offspring in rodent models of maternal stress experience [1, 9]. While the exact mechanisms by which environmental perturbations contribute to sex-specific vulnerability are not clear, mounting evidence suggests disruptions at the maternal-fetal interface [10, 11]. As the metabolic and immune orchestrator between mother and offspring, the developing placenta is highly sensitive to availability of circulating nutrients and metabolites [11–14]. Alterations to the maternal milieu during this critical window may fundamentally alter the structure and function of the placenta, resulting in downstream bottlenecks in placental nutrient and oxygen transport that ultimately contribute to elevated vulnerability and risk [11]. Sexually dimorphic patterns in energy substrate utilization have been observed during this window of early prenatal stress exposure, suggesting that the heightened male vulnerability to prenatal insults may be related to basal sex differences in metabolic requirements of male and female embryos [15, 16]. Further, the dynamic sex differences in placental transcriptomes may suggest that male and female placentas may respond to the same environmental insult through distinct pathways [17–19]. Indeed, exposure to chronic variable stressors during the first week of gestation resulted in dysregulation of placental genes involved in fatty acid transport and glucose metabolic processes in male, but not female, placentas in a mouse model of early prenatal stress (EPS) [18, 20, 21].

More recent work has demonstrated that maternal stress may influence the availability of nutrients essential for normal fetal neurodevelopment [22, 23]. Indeed, stress during pregnancy depletes maternal stores of polyunsaturated fatty acids (PUFAs) and decreases the amount of available PUFAs to the offspring [22, 23]. Moreover, maternal dietary deficiency of the omega-3 PUFA, docosahexaenoic acid (DHA), is associated with reduced placental weight, increased inflammation and oxidative damage in the placenta, and long-term dysregulation of stress neurocircuitry and behavior in offspring [22–27]. Conversely, supplementation of maternal dietary DHA stimulates angiogenesis resolves placental inflammation and oxidative stress, decreases pregnancy-associated risk, and improves behavioral outcomes in offspring [27–31]. Taken together, maternal supplementation of dietary DHA may influence key pathways that are vulnerable to disruption by maternal stress experience during pregnancy.

Therefore, to examine the potential interaction between maternal stress and diet on sex-specific expression of genes involved in placental metabolic pathways, we used a mouse model of early prenatal stress (EPS), in which male, but not female, offspring may show placental reprogramming, as well as increased stress sensitivity, cognitive dysfunction, and metabolic status in adulthood [21]. As maternal stress experience during pregnancy is associated with depleted maternal DHA stores, increased placental inflammation, and decreased DHA accumulation in the fetal brain, supplementation of dietary DHA to the dam may resolve stress effects on the placenta in a sex-specific manner. To examine this hypothesis, candidate genes involved in nutrient signaling and altered by EPS in a sex-specific manner were assessed in embryonic day (E) 12.5 placentas, with and without DHA supplementation [21]. In addition, to determine whether EPS and maternal dietary DHA supplementation exhibits an effect on early brain development, expression of the major neuroplasticity factors, brain-derived neurotrophic factor (BDNF), and cyclic AMP response element-binding (CREB) protein were assessed in embryonic day 12.5 fetal heads, a period of rapid neurodevelopment that is particularly sensitive to maternal stress and nutrient availability [32].

## Materials and methods

### Animals

Forty 6–8-week-old C57BL/6J females (P0 females) were purchased from Jackson Laboratories (Bar Harbor, Maine) and fed the control diet (detailed below) for at least 2 weeks while the mice habituated to the vivarium. Following habituation, animals were randomly placed on one of two diets: (i) the CTL diet with no additional DHA (CTL, *n* = 20) or (ii) the CTL diet supplemented with 1.0% wt DHA (1.0% DHA, *n* = 20). Additional detail on the experimental diets are provided below. For all experiments, the litter was the experimental unit such that only one male and one female were used for subsequent analysis. An important note on factors that impacted sample size: for each generation, a considerable number of animal are culled to limit the size of animal colonies such that each generation yields similar number of animals. Further, our breeding experiments yield a plug success rate of ~ 30–50% in C57Bl/6J strains and was subsequently confirmed by tracking body weight or at the time of collections. In addition, early prenatal stress model (see below) requires time-mating dams for both treatment groups. Collectively, these factors contributed significantly to the sample sizes in this study.

Animals were housed in clear polycarbonate cages (32 cm × 18 cm × 24 cm) provided with aspen bedding and a nestlet and maintained under standard conditions (25 ± 2 °C and 50% ± 10% humidity), with ad libitum access to water provided in glass bottles and diet specific to each treatment group, and on 12:12 h light cycle with lights on at 0600 CST. All experiments were approved by University of Missouri Animal Care and Use Committee and performed in accordance with National Institutes of Health Animal Care and Use Guidelines.

### Diet composition

The rodent diets started with the AIN-93G purified-diet profile (Dyets Inc., #110700) as the base. The AIN93G diet uses solely soybean oil as the source of fat, resulting in a 7:1 n-6:n-3 ratio. Early studies measuring in Western populations have reported ratios up to 50:1 n-6:n-3 ratio; however, more recent efforts have shown that the average n-6:n-3 ratio of individuals consuming a Western-type diet is closer to a 10:1 n-6:n-3 ratio [33–36]. In order to model the average fatty acid composition of a typical Western diet, we altered the AIN93G base profile by adding corn and soy oil at a 2:1 ratio to achieve a 10:1 n-6:n-3 ratio AIN93G diet (Dyets Inc., #103619). This modified diet served as the control and as the base for the experimental DHA diets. The CTL diet contained no preformed DHA but did contain sufficient amounts of alpha-linolenic acid (ALA, 18:3n-3) to meet normal brain DHA requirements [37]. An experimental diet was developed that was identical to the CTL diet except for the addition of 1.0% wt DHA (Dyets Inc., #103598). The DHA source used in this study (i.e., DHASCO algal oil) contained small amounts of other long-chain omega-3 PUFA ethyl esters and was provided as a generous gift from DSM Nutritional Products (Columbia, MD, USA). Both diets were stabilized against auto-oxidation by the addition of a synthetic antioxidant (i.e., 0.02 g tertiary-butylhydroquinone/100 g fat) [38]. Table 1 provides detailed fatty acid composition of the experimental diets.

**Table 1:** Fatty acid composition of diets

	Standard diet	DHA-enriched diet
	*(% by weight in diet)*	
**Fatty acid**		
16:0	0.77	0.39
18:0	0.24	0.16
18:1	1.76	1.00
18:2n6	3.86	4.01
18:3n3	0.37	0.02
20:5n3	nd	0.11
22:5n3	nd	0.26
22:6n3	nd	1.01
**Ratio n-6/n-3**	**10.4**	**2.9**

Note: *nd* not detectable

### Diet exposure protocol

Grain-based chow diets that are often utilized as gold standard laboratory chow diets show significant batch-to-batch variability and formulations differ between manufacturers [39, 40]. Given that systemic omega-3 PUFA levels are largely driven by dietary PUFA availability, variability in the source, amount, and composition of dietary fatty acids in these diet formulations may represent a potential confound [41]. To control for potential differences in the composition of dietary fatty acids that may result in physiologically relevant differences in omega-3 PUFA status, a multigenerational diet exposure protocol was applied to stabilize omega-3 PUFA levels and maintain the same levels across generations [42]. The founding population of females (i.e., P0) transitioned to a chow diet to either CTL or DHA-enriched diet. P0 females were mated with males consuming the CTL diet, and females were single-housed upon detecting a mating plug. Females remained on the assigned diet through gestation and lactation, and F1 offspring remained on the same maternal diet. Similar to the P0 breeding scheme, F1 females were mated with CTL-fed males, remained on the assigned diet during gestation and lactation, and F2 offspring were maintained the same diet as their mothers. To generate the F3 embryonic day (E)12.5 offspring used in the present study, F2 dams were mated to males consuming the CTL diet.

### Early prenatal stress

A chronic variable stress procedure was administered during gestational day 0.5–7.5 to F2 dams consuming either the CTL or DHA-enriched diets (early prenatal stress, EPS; *n* = 4 CTL diet, *n* = 4 1.0% DHA diet) for comparison to a control nonstressed (*n* = 3 CTL diet, *n* = 3 1.0% DHA diet) group. On detection of mating plug (e.g., E 0.5), pregnant mice assigned to the EPS group experienced each of the following stressors on a different day of the EPS period, as previously described [21]: 60 min (beginning at 11:00 AM) of fox odor exposure (1:5000 2,4,5-trimethylthiazole; Acros Organics), 15 min of restraint (beginning at 11:00 AM) in a mouse restraint tube, 36 h of constant light, novel noise (White Noise/Nature Sound-Sleep Machine; Brookstone) overnight, three cage changes (at 7:00 AM, 1:00 PM, and 5:00 PM) throughout the light cycle, overnight exposure to a novel object (twelve marbles of similar size), and saturated bedding (700 mL, 23 °C water) overnight. These stressors were previously shown to be nonhabituating and did not influence maternal food intake [21]. Together with the diet groups, there were four different groups in this study: (1) No EPS offspring exposed to the CTL diet (CTL-CTL); (2) EPS offspring exposed to the CTL diet (EPS-CTL); (3) No EPS offspring exposed to the 1.0% DHA diet (CTL-DHA); and (4) EPS offspring exposed the 1.0% DHA diet (EPS-DHA).

### Mouse tissue dissection

On gestational day 12.5, pregnant dams were weighed and rapidly decapitated by cervical dislocation. Litter characteristics such as intrauterine position, number of offspring, sex ratio, and resorption sites were noted. Corpora lutea from the left and right ovaries were collected and placed into ice-cold buffer, and total number of corpora lutea were counted. Fetal loss was calculated as the difference between number of corpora lutea and number of offspring.

The gravid uterus was transferred to a plastic culture dish containing ice cold 1 × PBS, and the dish was placed on ice. Embryo sites were dissected to separate individual embryos, which were placed in ice-cold 1× PBS filled wells to preserve RNA, DNA, and protein integrity. The amnion sac was removed, and the placenta was removed from the fetus. Tail snips from embryos were collected and used to identify sex of individual embryos by RT-PCR, using primers specific for Sry (5_-GAGTACAGGTGTGCAGCTCTA- 3’ and 5’-CAGCCCTACAGCCACATGAT-3’), as previously reported [17]. Thermal cycling conditions were 98 °C for 30 s, 54 °C for 40 s, and 72°C for 50 s for 30 cycles. Embryo and placental samples were rapidly frozen in liquid nitrogen and maintained in − 80 °C until RNA isolation. In order to control for the significant contribution of uterine horn laterality (e.g., weight differences due to implantation in the left vs. right uterine horn) and intrauterine position, an adjacent pair of male and female conceptuses with no overt signs of developmental delay (e.g., small size relative to litters and not neighboring a resorption site) in the left uterine horn were selected for subsequent analyses.

### Embryonic day 12.5 placental and fetal head RNA isolation and quantitative real-time PCR

Following sex determination of the placentas, RNA was isolated exactly as previously described by us [43]. Briefly, RNA isolation was conducted using the Qiagen RNAeasy Mini Kit (Qiagen). The TaqMan Fast Advanced Master Mix was used as the master mix for a 20 μL PCR reaction (ThermoFisher) and cycling conditions were as follows: Hold at 50 °C for 2 min; hold at 95 °C for 10 min; 30 cycles of 15 s at 95 °C followed by 60 s at 60 °C. Candidate genes were chosen based on previous reports of sex-specific dysregulation of these placental genes following EPS [18, 21]. Changes in hypoxia-inducible factor 3a (Mm00469375_m1; NM_001162950.1), O-GlcNAc transferase (Mm00507317_m1; NM_139144.3), vesicular endothelial growth factor A (Mm01281449_m1; NM_001025250.3), insulin-like growth factor-binding protein 1 (Mm00515154_m1; NM_008341.4), glucose transpoter 4 (Mm01245502_m1; NM_009294.2), DNA methyltransferase 1 (Mm01151063_m1; NM_001199431.1), and peroxisome proliferator-activated receptor alpha (Mm00440939_m1; NM_001113418.1) were measured. Tissue samples were analyzed in at least triplicates with a critical threshold standard deviation of 0.5 within each triplicate. The threshold cycle was normalized to the housekeeping gene β-actin (Actb; NM_007393.3;Mm00607939_s1) using an ABI Real-time 7500 system (Applied Biosystems, Waltham, MA). The expression level for each gene in each tissue was calculated using the comparative CT method. In brief, the cycle number at threshold was used for calculations of relative amount of mRNA molecules. The CT value of each target gene was normalized by subtraction of the CT value from β-actin. This value is defined as the ΔCT. While it is likely that EPS and control animals within a diet are likely to differ, the central hypothesis of the present study is that maternal DHA enrichment will buffer EPS-related programing relative to EPS animals exposed to a control diet. As a result, relative quantitative change was calculated using the formula 2^−(ΔCT EPSCTL-ΔCT EPSDHA)^ (*n* = 3–5 mice/sex/diet/stress).

### Statistical analysis

Litter characteristics data is presented as mean (± SD), while data on placenta and fetal weight and qPCR data is presented as mean (± SEM) and analyzed within the R environment using nlme and lattice packages [44–46]. Litter characteristics, placenta and embryo weights, and qPCR results from the placenta and embryonic tissue samples were analyzed with a 2 (stress) × 2 (sex) × 2 (diet) ANOVA. Tukey’s HSD post hoc comparison was used for all group-level contrasts.

## Results

### Effect of EPS and maternal diet on litter characteristics

To determine whether prenatal stress, DHA enrichment, or its interaction alter litter characteristics, we assessed for the combined treatment effects on litter size, fetal loss, and sex ratio. Table 2 shows litter characteristics. An independent *t* test revealed no difference in fetal loss (e.g., difference between corpora lutea and number of offspring present at time of collection) between EPS (M = 4, SD = 3.08) and non-EPS dams (M = 1, SD = 1) consuming the CTL diet (t_6_ = − 1.59, *p* = 0.16). Similarly, there was no difference in fetal loss between EPS (M = 0.50, SD = 1.0) and non-EPS dams (M = 0, SD = 0) consuming the DHA-enriched diet (t_6_ = − 0.845, *p* = 0.44). There was, however, a main effect of diet on fetal loss (*F*_1,11_ = 6.40, *p* = 0.028), with CTL diet females exhibiting higher fetal loss than DHA-enriched diet females (*p* = 0.041), but the main effect of EPS and the diet x EPS interaction were not significant (*p* values = 0.11 and 0.26, respectively). No other litter characteristics were different across diet or stress treatment.

**Table 2:** Maternal and litter characteristics by diet and early prenatal stress

Dams	Litter size	Males	Females	Corpora lutea	Fetal* loss	Resorptions	Sex ratio
CTL–No EPS	7 ± 1.63	4.66 ± 1.25	2.33 ± 0.47	8 ± 1.41	1 ± 0.82	1 ± 1.414	0.66
CTL–EPS	5 ± 2.53	2.8 ± 1.33	2.2 ± 1.72	9 ± 2.76	4 ± 2.76	1.6 ±0.8	0.64
No EPS–DHA	7.66 ± 0.47	4.33 ± 1.25	3.33 ± 0.94	7.66 ± 0.47	0	0.333 ± 0.47	0.56
EPS–DHA	7.5 ± 0.5	5.25 ± 0.43	2.25 ± 0.83	8 ± 0.71	0.5 ± 0.87	0.5 ± 0.87	0.71

Data presented as Mean ± SD. Fetal loss was quantified as the difference between litter size and number of corpus lutea

*Denotes a significant main effect of diet on fetal loss (*p =* 0.041), but diet*EPS interaction was not significant. Please refer to the “Effect of EPS and maternal diet on litter characteristics” section in the main text

To determine whether maternal diet and stress influenced placenta weight, samples were collected and weighed at E12.5. Analysis showed a main effect of diet (*F*_1,22_ = 7.56, *p =* 0.012; CTL = 52 ± 1 mg; DHA = 60 ± 3, mean ± SD) and a trending sex × EPS interaction (*F*_1,22_ = 3.95, *p =* 0.0505) on placental weight. However, we failed to detect diet × EPS (*p* = 0.28) nor sex × diet × EPS interactions (*p* = 0.14) on placental weight. The DHA-enriched diet increased placental weight compared with CTL diet placentas (*p =* 0.012). Further, we detected a trend for male placentas exposed to early prenatal stress weighing less than stress-exposed female placentas (*p =* 0.052). Additional examination of the diet × EPS interactions revealed that male placentas from dams consuming the DHA-enriched diet showed increased placental weight relative to CTL males (*p* = 0.049). No other pairwise comparisons were significant.

We next determined whether maternal diet and stress influenced embryo weight. Analysis revealed a main effect of diet (*F*_1,22_ = 5.26, *p* = 0.032; CTL = 39 ± 2 mg; DHA = 43 ± 4, mean ± SD) and EPS (*F*_1,22_ = 8.03, *p* = 0.009; CTL = 39 ± 2 mg; EPS = 36 ± 3, mean ± SD) on embryo weight, but there was no main effect of sex (*p =*0.51) or any significant interactions; diet × EPS (*p =* 0.16), sex × EPS (*p =* 0.78), diet × sex × EPS (*p* = 0.42). DHA-enriched embryos weighed more than CTL diet embryos (*p =* 0.032), and EPS decreased embryo weight (*p* = 0.009). Although the diet × EPS interaction was not significant, post hoc contrasts revealed a trend for exposure to EPS decreased embryo weight in animals exposed to the CTL diet relative to non-stressed animals consuming the same diet (*p =* 0.051), whereas no such difference emerged in the DHA diet animals (*p* = 0.92). No other pairwise comparisons were significant.

### Effect of EPS and maternal diet on placental gene expression

To determine whether DHA enrichment impacts EPS sex-specific disruption, control and EPS E12.5 placentas exposed to the diets were examined using a series of candidate genes, specifically, genes that have been previously shown to exhibit male-specific disruption in placental gene expression and are associated with increased disease risk in males in adulthood.

**Fig. 1:**
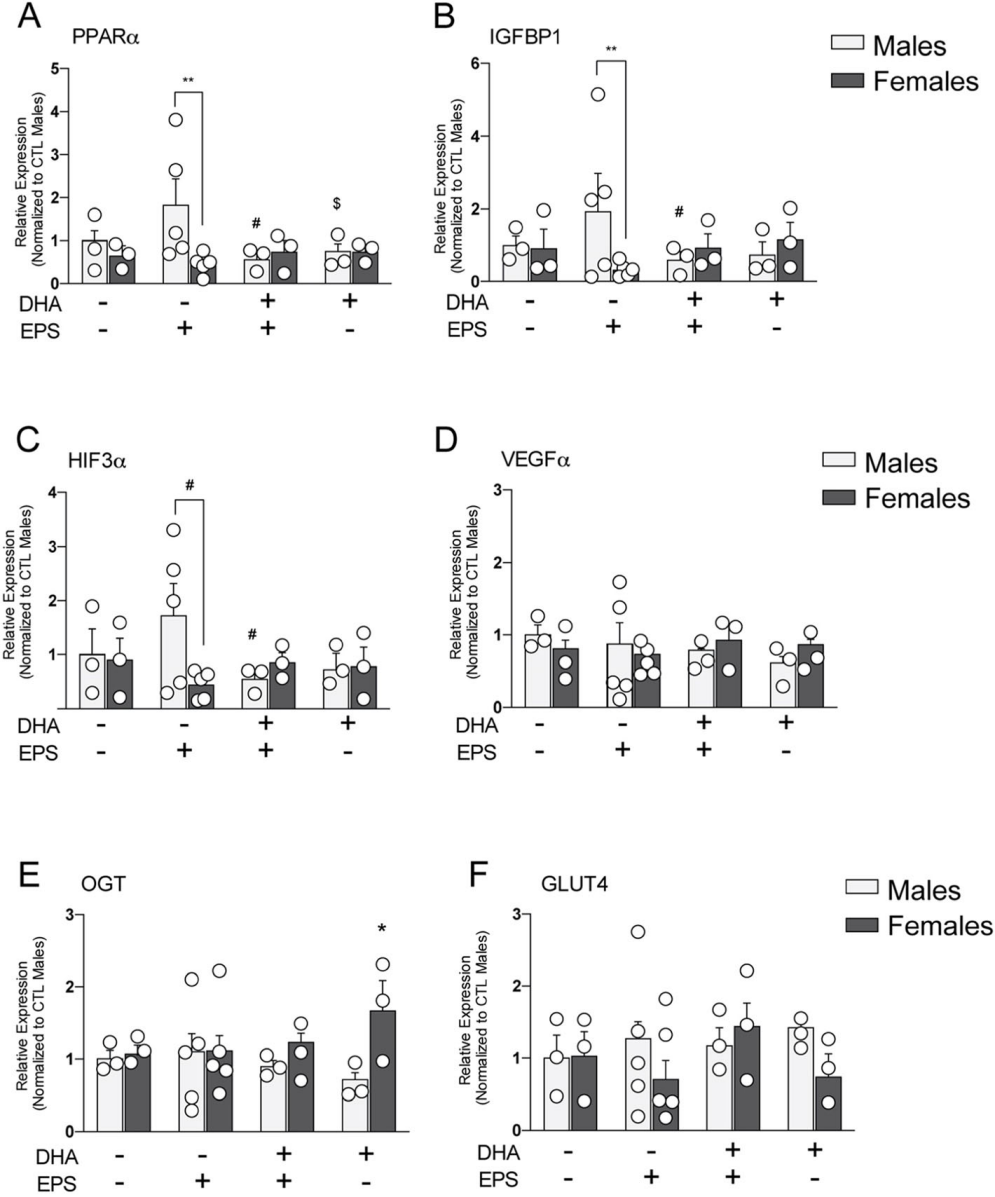
Effects of DHA enrichment and EPS on expression of genes that are critical for vascularization and nutrient transport in male and female placentas (please refer to the “Results” section for additional details). **a** There was a diet × sex interaction of peroxisome proliferator-activated receptor alpha (PPARα) expression (*F*_1,10_ = 12.34, *p* = 0.006). Post hoc analysis revealed PPARα expression was upregulated in EPS-CTL diet male placentas relative to EPS-CTL female placentas (*p* = 0.042, denoted in the figure as the **). PPARα was downregulated in EPS-DHA diet male placentas relative to EPS-CTL diet male placentas (*p* = 0.038, denoted in the figure as the #). PPARα was downregulated in CTL-DHA diet male placentas relative to EPS-CTL male placentas (*p* < 0.05, denoted in the figure as the $). **b** There was a diet × sex interaction in expression of insulin-like growth factor-binding protein1 (IGFBP1) in the placenta (*F*_1,10_ = 6.17, *p* = 0.032). Post oc analysis revealed that IGFBP1 was upregulated in EPS-CTL males compared with EPS-CTL female placentas (*p* < 0.01). IGFBP1 was upregulated in EPS-CTL males compared with EPS-DHA male placentas (*p* < 0.05, denoted in the figure as the #). **p < 0.01. **c** There was a diet × sex interaction on hypoxia inducible factor 3a (HIF3α) expression (*F*_2,14_ = 10.93, *p* = 0.008). Post hoc analysis revealed a trend towards HIF3α upregulation in EPS-CTL diet male placentas relative to EPS-CTL diet female placentas (*p* = 0.05, denoted in the figure as #). Additionally, there was a trend for upregulation of HIF3α expression in EPS-CTL male placenta relative to EPS-DHA male placenta (*p* = 0.062, denoted in the figure as #). **d** There was a trending main effect of sex on expression of vascular endothelial growth factor A (VEGFA) (*F*_2,14_ = 4.34, *p* = 0.053), whereby females exhibited upregulation compared with males. No other significant pairwise comparisons were detected. **e**, **f** No significant effects were observed in the gene expression of **e** OGT and **f** GLUT4 across treatment groups (all *p* values > 0.05). *n* = 3–5 animals/sex/diet/stress treatment. **p < 0.01. Data represented as mean ± SEM with individual data points overlaid

There was a diet × sex interaction of peroxisome proliferator-activated receptor alpha (PPARα) expression (*F*_1,10_ = 12.34, *p* = 0.006) (Fig. 1a). PPARα expression was upregulated in EPS-CTL diet male placentas relative to EPS-CTL female placentas (*p* = 0.042), but there was no sex difference in PPARα transcription in EPS-DHA diet placentas (*p* = 0.36). PPARα expression did not differ across EPS female groups (*p* = 0.35). Surprisingly, PPARα was downregulated in EPS-DHA diet male placentas relative to EPS-CTL diet male placentas (*p* = 0.038), which accounts for the lack of a difference comparing EPS-DHA diet male and female placentas.

There was a diet × sex interaction in expression of insulin-like growth factor-binding protein1 (IGFBP1) in the placenta (*F*_1,10_ = 6.17, *p* = 0.032) (Fig. 1b). The interaction emerged because IGFBP1 was upregulated in EPS-DHA females as compared to males, and upregulated in EPS-CTL males compared to females; no other pairwise contrasts were significant. There was a diet × sex interaction on hypoxia-inducible factor 3a (HIF3α) expression (*F*_2,14_ = 10.93, *p* = 0.008) (Fig. 1c). HIF3α expression was trending towards upregulation in EPS-CTL diet male placentas relative to EPS-CTL diet female placentas (*p* = 0.05), but there was no sex difference in HIF3α expression in EPS placentas exposed to a DHA-enriched diet (*p =* 0.41). HIF3α expression did not differ across the EPS female groups (*p =* 0.34), but there was a trend for upregulation of HIF3α expression in EPS-CTL male placenta relative to EPS-DHA male placenta (*p* = 0.062).

There was a trending main effect of sex on expression of vascular endothelial growth factor A (VEGFA) (*F*_2,14_ = 4.34, *p* = 0.053), whereby females exhibited upregulation compared with males (Fig. 1d). Neither the main effect of diet (*p* = 0.58) nor the sex × diet interaction (*p* = 0.68) was significant. However, expression of O-linked N-acetylglucosamine transferase (OGT) was not related to sex (*p* = 0.16) or diet (*p* = 0.76) or the interaction (*p* = 0.36) (Fig. 1e). Similarly, expression of glucose transporter 4 (GLUT4) in the placenta was not related to sex (*p* = 0.71), diet (*p* = 0.22), or the interaction (*p* = 0.13) (Fig. 1f).

### Analysis of BDNF and CREB in E12.5 fetal heads

As placental gene expression patterns may influence development of tissues such as the brain, we also examined the impact of diet and EPS on gene expression in E12.5 whole brain samples. However, expression of genes encoding brain derived neurotrophic factor (BDNF) was not related to sex (*p* = 0.16) or EPS (*p* = 0.73) or its interaction (*p = 0.*48) (no EPS = 1 ± 0.23; EPS = 0.92 ± 0.41, group-level mean ± SEM). Expression of cAMP response-binding element protein (CREB) was not related to sex (*p* = 0.30) or EPS (*p* = 0.36) or its interaction (*p* = 0.31) (no EPS = 1 ± 0.19; EPS = 1.17 ± 0.23, group-level mean ± SEM). Based on the lack of differences between EPS and non-EPS groups exposed to the CTL diet, we did not measure BDNF and CREB expression in DHA diet cohorts.

## Discussion

Collectively, this study yielded two results regarding the interaction between maternal stress and dietary DHA enrichment in embryonic day 12.5 conceptuses. First, maternal stress during the first week of gestation appeared to influence the composition of the litter and gene expression patterns in the placenta, with offspring sex appearing to predominantly determine the magnitude of disruption. Second, a maternal diet enriched with preformed DHA during periods of high stress shows partial rescue of stress-dependent dysregulation of gene expression in the placenta.

The observation that the placenta responds to maternal diet is not surprising given its specialized metabolic niche that is particularly sensitive to maternal resource availability. Maternal malnutrition during pregnancy, presumably through constrained exchange of maternal nutrients across the placenta, exerts long-term changes in morbidity and mortality, growth trajectory, and increased disease risk in adulthood [47]. Maternal diet also appears to exhibit contrasting programing on the placental transcriptome based on offspring sex, consistent with maternal diet influencing resource exchange through the placenta in a sex-specific manner [19].

In the present study, maternal diet was associated with some aspects of growth and development. Mothers consuming the DHA-enriched diet exhibited lower fetal loss relative to offspring of CTL-diet mothers, suggesting that the availability of DHA in maternal diet may be critical to fetal development at this development window. Although there were no sex differences in placental weight within the same maternal diet, DHA enrichment increased male placental weight relative to that of males in the CTL diet group. Previous reports have shown that placental weight is associated with offspring size, which may be attributed to differential resource demand for growth-related nutrients and downstream consequences on newborn size [48]. Indeed, DHA enrichment increased embryo weight at E12.5 relative to offspring exposed to CTL diet, and this effect was independent number of offspring given no diet-related differences in litter size. These results must be interpreted with caution, however, as high rates of infanticide have been reported for the C57Bl/6J strain used in this study, and, as a result, increased offspring size at E12.5 may not reflect litter size or survival rates following birth [49].

Similar to maternal nutrition status, maternal stress during pregnancy has direct consequences on offspring growth and development in humans, and later phenotypic outcomes in adulthood across multiple species, including increased risk for neurodevelopmental and neuropsychiatric disorders in humans [10]. Consistent with previous results, we observed that maternal stress trended toward reduction in male placental weight, with no effect of prenatal stress on female placental weight [50]. To assess the potential factors involved in the sex differences in placental and embryo size, we measured the expression of placental genes previously reported to be sensitive to sex-specific disruption following EPS. Similar to previous reports, EPS resulted in male-specific upregulation of placental HIF3α and PPARα, with no effect on females [21]. However, there was no sex or EPS-related difference in expression of OGT, IGFBP1 or GLUT4 [18, 21]. The discrepancy between previous and current study is likely related to the differences in genetic background of mouse strains (C57Bl/6J vs. mixed background of C57Bl/6J*129), which exhibit differential sensitivity to stress [51]. In addition, differences in the sensitivity of detection methods that were used to measure gene expression (i.e., SYBR-based PCR arrays compared with TaqMan gene expression assay in the present study) may provide another additional explanation for differences in the results between studies. Nevertheless, our results showed that DHA-enrichment also exhibited no effect on placental expression in either EPS-exposed or control females, further suggesting male-specific vulnerability to early prenatal stress may be buffered by maternal diet.

Based on the observation that maternal diet and maternal stress result in similar sex-specific changes to placental and embryo size, we examined whether maternal diet and maternal stress converge upon similar transcriptional pathways. DHA enrichment reversed the EPS induced male-specific upregulation of placental HIF3α and PPARα back to comparable levels of males that were not exposed to early prenatal stress. Low oxygen conditions activate a cascade of physiological response that includes the upregulation of large class of hypoxia inducible factor [52–54]. HIF proteins control a broad family of genes, including VEGFA, a canonical regulator of angiogenesis that is highly sensitive to hypoxic conditions [52–54]. EPS increased expression of HIF3α but did not result in a parallel EPS-dependent upregulation of VEGFA. The inability of HIF3α to increase expression of VEGFA may be related to the unique structural properties of HIF3α. In contrast to family protein members HIF1 and HIF2, HIF3α lacks a C-terminal activation domain required for co-activator binding, and, as a result, is unable to recruit co-transcriptional regulators and basal transcriptional machinery to gene targets, including VEGFA [52–54].

An alternative interpretation to the finding that EPS induces male-specific reprogramming of candidate genes in the placenta would be that this represents a sex-specific adaptation to increase resource transfer during periods of stress, and, as a result, DHA-enrichment is hampering this adaptation [12, 55]. Such an alternative hypothesis would predict either no difference in placental and embryo weight for EPS and non-stress animals exposed to the CTL diet or a negative effect of DHA diet on placental and embryo weight. The present results show the opposite trend: DHA enrichment increases placental and embryo weight while concomitantly decreasing expression of genes that are normally expressed in low oxygen or constrained nutrient conditions.

Nevertheless, the present results can be readily understood within maternal life history trade-offs [56]. During pregnancy, mothers require resources to meet both maternal and offspring requirements. However, environmental cues, such as malnutrition and stress, may decrease the optimal resource transfer from mothers to offspring in a way that maximizes maternal reproductive success at the detriment to offspring. Sex-specific reductions in male, but not female, placenta and embryo in EPS-exposed animals are consistent with reduction of maternal investment in male relative to female offspring. From an evolutionary perspective, the shift in maternal resource allocation may be related to sex differences in reproductive payoffs for mothers; specifically, smaller, less fit males are less likely to reproduce as adults than their sisters [57]. Indeed, rescue of this sex-specific vulnerability with dietary DHA enrichment may suggest that EPS-dependent deficiency of nutrient and oxygen transfer may trigger this shift in maternal investment. The goal of future studies should focus on whether DHA enrichment buffers from prenatal stress-dependent deficits in adulthood and on identifying sex- and diet-dependent broad programmatic pathways following exposure to prenatal stress through deeper characterization of the phenotype and by leveraging protein-level analysis and bulk transcriptome profiling approaches. Moreover, the possibility that DHA enrichment may ameliorate the effects of stress across pregnancy and into early life is an exciting avenue for future research.

## Perspectives and significance

Taken together, the results of this exploratory study show that maternal consumption of DHA-enriched diet during chronic stress exposure during the first week of pregnancy may influence gene expression patterns of the placenta in a sex-specific manner. Additional follow-up studies are now needed to follow-up what is suggested in this initial exploratory study, and to better understand the complex cellular and molecular mechanisms linking maternal diet consumption, chronic stress during pregnancy, placental gene expression, and lasting health outcomes in offspring. Since the entire placenta from these samples was used in the above described analysis, measuring protein levels of these genes was not possible for this specific study. Future studies will need to focus on cell-type specific protein levels of the genes that were affected in these exploratory studies. Cell-type specific expression of the genes is critical. To this point, Vento-Tormo et al. [58] previously published a single-cell reconstruction of the maternal-fetal interface that includes analysis of placenta samples, for example, showing that VEGFA expression is largely localized to perivascular cells. For instance, previous work in this model has shown that early prenatal stress is associated with lasting disruption to the expression of glucocorticoid receptor expression in the brain, inflammation, and excessive corticosterone production following acute stress exposure [21]. The possibility that consumption of a DHA-enriched diet early in pregnancy may protect against maternal stress-mediated effects on proteomics, immunity, glucocorticoid receptor expression, interactions with estrogen, and stress responsivity in adult offspring are the focus of follow-up studies.  This is of additional interest given the finding that DHA reverses social behavior and repetitive behavior changes in adult offspring induced by prenatal stress exposure later in pregnancy in genetically stress susceptible dams, and additionally reverses the increases in striatal dopamine in these offspring exposed to prenatal stress [59]. Given that consumption of a DHA-enriched diet may influence additional tissues, further evaluation will be needed to determine the long-term impact on the brain as well as on other systems by leveraging bulk and single-cell sequencing approaches that may identify programmatic effects of maternal stress and diet in males and females at a cell-type specific resolution. Since gene expression is highly dynamic during development, changes in VEGFA and CREB, for example, while not observed in this study, may be detected at other timepoints. Lastly, given that dietary components are highly modifiable, these studies may provide additional support for interventions targeted towards increasing micronutrient availability of preformed DHA in women with a history of chronic stress or trauma.

## Data Availability

No custom code or software was used for the analysis discussed in this manuscript. Please contact author for data requests.

## Abbreviations

CTL: Control
EPS: Early prenatal stress
DHA: Docosahexaenoic acid
qPCR: Quantitative polymerase chain reaction
ANOVA: Analysis of variance
PPARα: Peroxisome proliferator activated receptor alpha
GLUT4: Glucose transporter 4
OGT: O-linked N-acetylglucosamine transferase
HIF3A: Hypoxia-inducible factor 3 alpha
IGFBP1: Insulin-like growth factor-binding protein 1
VEGFA: Vascular endothelial growth factor alpha
CREB: Cyclic AMP response element-binding protein
